# Protective role of zeaxanthin on acrylamide-induced neurotoxicity in Wistar rats

**DOI:** 10.22038/AJP.2024.24950

**Published:** 2025

**Authors:** Zoha Mortazavi, Mahboobeh Ghasemzadeh Rahbardar, Soghra Mehri, Hossein Hosseinzadeh

**Affiliations:** 1 *School of Pharmacy, Mashhad University of Medical Sciences, Mashhad, Iran*; 2 *Pharmaceutical Research Center, Pharmaceutical Technology Institute, Mashhad University of Medical Sciences, Mashhad, Iran*; 3 *Department of Pharmacodynamics and Toxicology, School of Pharmacy, Mashhad University of Medical Sciences, Mashhad, Iran*

**Keywords:** Acrylamide, Zeaxanthin, Antioxidant, Anti-Inflammatory, Apoptosis, Neuroprotective agents

## Abstract

**Objective:**

The Maillard reaction generates acrylamide (ACR), a toxic compound commonly found in laboratory and industrial settings. ACR exposure, both short-term and long-term, can damage various organs, notably the central nervous system, through oxidative stress, inflammation, and apoptosis. This study explores the potential neuroprotective effects of zeaxanthin (ZEA), known for its antioxidant, anti-inflammatory, and anti-apoptotic properties, against ACR-induced toxicity in the rat cerebral cortex.

**Materials and Methods:**

Rats were subjected to ACR exposure (50 mg/kg, intraperitoneal injection) for 11 days and subsequently, treated with ZEA (20-80 mg/kg, intragastric gavage) for either 11 or 20 days to assess both preventive and therapeutic effects. Locomotor behavior was evaluated using a gait score test, while biochemical analyses measured malondialdehyde (MDA) and glutathione (GSH) levels, inflammatory markers interleukin-1 beta (IL-1β), and tumor necrosis factor-alpha (TNF-α), and apoptotic markers (cleaved caspase-3) in the cerebral cortex.

**Results:**

ACR exposure impaired locomotion in the animals, but ZEA treatment significantly improved gait scores when administered preventatively (from days 6-11) or therapeutically (from days 6-20). ACR also led to increased MDA levels and depleted GSH content in brain tissue, and it elevated IL-1β, TNF-α, and cleaved caspase-3 in the cerebral cortex. However, ZEA supplementation, along with vitamin E, effectively reversed these alterations compared to the ACR-exposed group.

**Conclusion:**

In conclusion, ZEA demonstrates both preventive and therapeutic effects against ACR-induced neurotoxicity. These findings suggest that ZEA could serve as an effective preventive agent by countering ACR-induced damage through its antioxidant, anti-inflammatory, and anti-apoptotic mechanisms.

## Introduction

Acrylamide (ACR), also known as 2-propenamide, is a colorless, water-soluble crystalline powder with significant toxicity (Riboldi et al., 2014). It is primarily formed during the cooking process of carbohydrate-rich foods at high temperatures and is commonly found in various processed foods like French fries, chips, baked bread, and barbecued meat (Kopanska et al., 2018). Beyond its presence in food, ACR serves diverse industrial and laboratory purposes, including the synthesis of electrophoresis gel, water purification, soil and waste treatment, and building sealing. It is also found in materials such as wood, paper, cosmetics, and cigarette smoke (Matoso et al., 2019). According to the Joint FAO/WHO Expert Committee on Food Additives (JECFA), the average daily exposure to ACR through regular dietary intake is estimated to be 0.6 μg/kg for adults and 1-4 μg/kg for children (Koszucka et al., 2020). The potential toxic effects of ACR on the reproductive and nervous systems, as well as its suspected genotoxicity and carcinogenicity upon exposure, have been extensively studied (Exon, 2006; Tandisehpanah et al., 2022). Notably, concerns about ACR's neurotoxic effects were first raised in 1997, highlighting the significance of its potential toxicity (Matoso et al., 2019).

Contrary to ACR monomers, their polymers are not toxic. Furthermore, despite being a 2-alkene, ACR is less electrophile and has a lower protein binding capacity than acrolein, a compound from the same family. These properties make ACR able to distribute significantly into the nervous system (Erkekoglu and Baydar, 2014). People who were exposed occupationally to ACR manifested symptoms of peripheral neuropathy (pain, paresthesia, and skeletal weakness), ataxia and skin irritation (Pennisi et al., 2013). Different mechanisms and cellular and molecular signaling pathways are responsible for ACR toxicity. ACR raised oxidative stress by elevating reactive oxygen species (ROS) levels, inducing lipid peroxidation and formation of malondialdehyde (MDA). The compound can also meddle with antioxidant enzyme activity, depressing levels of reduced glutathione (GSH) in a dose-dependent manner in various organs (Catalgol et al., 2009; Pan et al., 2018; Prasad, 2012; Yousef et al., 2006) especially in the nervous system (Imam et al., 2019; Pan et al., 2015). ACR has also caused inflammation leading to cellular death through increasing gene expression of tumor necrosis factor-alpha (TNF-α) and interleukin-1 beta (IL-1β) (Acaroz et al., 2018; Koszucka et al., 2020). These inflammatory properties of ACR in the nervous system have been observed in different animal models (Abdel-Daim et al., 2020; Elhelaly et al., 2019). Moreover, apoptosis and mitochondrial dysfunction are the other reported mechanisms that play an important role in ACR toxicity (Liu et al., 2015; Nowak et al., 2020). Additionally, according to animal experiments, ACR treatment caused a rise in the levels of apoptosis markers such as caspase-3 and Bax in cerebral tissues (Elblehi et al., 2020; Ghasemzadeh Rahbardar et al., 2022). 

Several medicinal plants or their constituents such as carnosic acid (Ghasemzadeh Rahbardar et al., 2022), crocin (Mehri et al., 2015), thymoquinone (Mehri et al., 2014; Tabeshpour et al., 2020) and ellagic acid (Goudarzi et al., 2019) have been shown protective effects against neurotoxicity induced by ACR. 

Zeaxanthin (ZEA) (3, 3’-dihydroxy-𝛽-carotene, C40H56O2) is a carotenoid xanthophyll found in egg yolk, some orange or yellow fruits, and vegetables like corn and carrots (Edwards, 2016; Murillo et al., 2019). JECFA considers a daily intake of up to 2 mg/kg in humans to be safe. However, it should be noted that administration of even 1 g/kg/day of ZEA in mice and rats for long periods revealed no specific adverse effects in organs and was tolerated well (Edwards, 2016).

ZEA has demonstrated significant therapeutic potential in various conditions including colitis (El-Akabawy and El-Sherif, 2019), hepatic injury (Li et al., 2017), metabolic syndrome, and cardiovascular diseases (Kou et al., 2017; Murillo et al., 2019) across different studies. Both animal and clinical research have highlighted ZEA's antioxidative properties, attributed to its ability to inhibit ROS formation, neutralize free radicals, and reduce MDA levels. Additionally, ZEA enhances cellular antioxidant defenses by increasing glutathione concentration and promoting the activity of enzymes such as superoxide dismutase and catalase (El-Akabawy, 2019; Murillo et al., 2019). Moreover, ZEA has been effective in suppressing inflammation (Firdous et al., 2015; El-Akabawy and El-Sherif, 2019; Li et al., 2022) and apoptosis (Ying et al., 2017) in cerebral tissues, through decreasing TNF-α and IL-1β content, and depressing amounts of apoptotic proteins such as caspase-3-cleaved, consequently improved cognitive function (Stringham et al., 2019) and alleviated depression and anxiety in animal studies (Zhou et al., 2018). 

With the key involvement of oxidative damage, inflammation, and apoptosis in ACR-induced neurotoxicity, and considering ZEA's capacity to combat radicals, improve cell survival, and reduce pro-inflammatory cytokines, this study aimed to explore ZEA's neuroprotective effects against ACR-induced neurotoxicity in rats, focusing on these underlying mechanisms.

## Materials and Methods

### Materials

Acrylamide from Merck (Germany), thiobarbituric acid (TBA) and 5, 5 ′ di thiobis-(2-nitrobenzoic acid) (DTNB) were obtained from Sigma-Aldrich (Germany). Polyvinylidene fluoride (PVDF) membrane was provided by BioRad (USA). Rabbit polyclonal antibody against IL-1β was purchased from Abcam (UK), rabbit monoclonal antibody against TNF-α, rabbit monoclonal antibody against caspase-3, mouse monoclonal antibody against β-actin, anti-mouse IgG antibody labeled with horseradish and anti-rabbit IgG antibody labeled with horseradish peroxidase were obtained from Cell Signaling (USA). 

### Animals

Male Wistar rats weighing between 230 and 250 grams were obtained from the School of Pharmacy, Mashhad University of Medical Sciences. They were housed in contamination-free colony rooms under a 12:12 hr light-dark cycle at a constant temperature of 21±2°C, with free access to food and water *ad libitum*. The study was conducted in accordance with ethical principles and national norms and standards for medical research in Iran, approved by the Research Ethics Committee of the Mashhad University of Medical Sciences under approval ID REC.PHARMACY.MUMS.IR.1398. 026.

### Experimental protocol

To induce the anticipated behavioral neurotoxicity, the dose, duration, and method of administering ACR were established based on previous studies with similar objectives (Mehri et al., 2016; Mohammadzadeh, 2018). In the present research, rats were sorted at random into 11 groups (n=6 in each group) and treatment was given as follows: 

(1) Control, normal saline intraperitoneal (i.p.) injection on days 1-11,

 (2) Control, normal saline i.p. injection on days 1-20, 

(3) ACR (50 mg/kg i.p.) administration on days 1-11, 

(4) ACR (50 mg/kg i.p.) administration on days 1-11 and saline i.p. injection on days 12-20, 

 (5) ACR (50 mg/kg i.p.) + ZEA (20 mg/kg) intragastric gavage (i.g.) on days 1-11, 

(6) ACR (50 mg/kg i.p.) + ZEA (40 mg/kg i.g.) on days 1-11 (Zhou et al., 2017), 

(7) ACR (50 mg/kg i.p.) + ZEA (80 mg/kg i.g.) on days 1-11 (Zhou et al., 2017),

(8) ACR (50 mg/kg i.p.) on days 1-11 and ZEA (80 mg/kg i.g.) on days 6-11, 

(9) ACR (50 mg/kg i.p.) on days 1-11 and ZEA (80 mg/kg i.g.) on days 6-20, 

(10) ZEA (80 mg/kg i.g.) on days 1-11,

(11) ACR (50 mg/kg i.p.) + vitamin E ( Vit E 200 mg/kg i.p. every other day) on days 1-11 (Mehri et al., 2015).

It should be noted that ACR was dissolved in normal saline and ZEA was dissolved in corn oil.

### The behavioral index (gait score) examination

After completing the 11-day treatment, the gait score of each rat was assessed. Twenty days after the initiation of the protocol, the assessment was performed on rats from groups 2, 4, and 9. Rats were put on plexiglass and allowed to move freely. Each animal was monitored for 3 min. The subjective gait score was assigned in the four-level score including (LoPachin 2005): 

1. Unaffected gait, or normal gait; 2. Slightly affected gait (foot splay and mild weakness of the hind limbs); 3. Moderately affected gait, including splayed feet, moderate hindlimb weakness, and spreading of the feet when walking; and 4. A severely affected gait characterized by strong hindlimb weakness and dragging of the hindlimbs. 

### Tissue sampling

After assessing the gait scores, rats were sacrificed with guillotine. No anesthetic or analgesic was used due to the possibility of exerting any unwanted neurological changes. Then cerebral cortexes were obtained and the samples were then snap-frozen in liquid nitrogen and after that stored at -80° C until analyzed.

### Determination of the MDA concentration in the cerebral cortex

To assess MDA content as a crucial indicator of lipid peroxidation in brain tissues, samples were homogenized with KCl to create a 10% homogenate. Subsequently, 3 ml of 1% phosphoric acid and 1 ml of 0.6% TBA were added to 0.5 ml of the homogenate. The mixture was then placed in a boiling water bath. After 45 min, the mixtures were gradually cooled, and 4 ml of n-butanol was added. Each sample was vortex-mixed for 1 min. The mixture was then centrifuged at 3500 g for 20 min. The organic layer was collected and transferred to new test tubes. The absorbance of the pink chromogen formed by the reaction of MDA and TBA was measured at 532 nm. The intensity of the color is directly proportional to MDA levels, serving as a marker for lipid peroxidation in brain tissues (Uchiyama and Mihara 1978).

### Measuring the amount of GSH in the cerebral cortex

The principle of GSH content assay is to measure the yellow chromophore produced through GSH and DTNB reaction. For this purpose, 10% homogenates of samples in phosphate buffer (pH 7.4) were mixed with tricarboxylic acid (TCA) in equal proportions and then, centrifuged at 3000 g. The upper layer (0.5 ml) was removed and added to 7.5 ml of phosphate buffer (pH 8.0). After adding 0.5 ml of DTNBT the absorbance was recorded at 412 nm. Levels of GSH were considered as nmol/g tissue (Araujo et al., 2008).

### Western blot analysis

Proteins were extracted from cerebral cortex tissue and were homogenized by adding a homogenizing buffer. Total protein contents were calculated by Bradford assay using bovine serum albumin (BSA) as standard (Bradford 1976). All samples were diluted with homogenizing buffer to have equal protein concentration. In the next step, sodium dodecyl sulfate (SDS) was added and mixtures were heated at 95°C for 5 min. Aliquots were stored at -80ᵒC until use. After polyacrylamide gel electrophoresis, proteins were transformed into a PVDF membrane. Subsequently, membranes were blocked by 5% skim milk in 5 ml tris-buffered saline with 0.1% Tween® 20 detergent (TBST) for 2 hr at room temperature. After blocking, membranes were incubated for 2 hr on a rocker with rabbit primary polyclonal antibody TNF-α (1:1000, Cell Signaling #3707), rabbit primary monoclonal antibody IL-1β (1:1000, Abcam #9722), caspase-3 (1:1000, Cell Signaling #9665) and mouse primary polyclonal antibody β-actin (1:1000, Cell Signaling #3700). Following washing of primary antibodies, membranes were incubated with mouse or rabbit horseradish peroxidase-conjugate anti-IgG (1:3000, Cell Signaling #7076; and 1:3000 Cell Signaling #7074; 1:3000, respectively) in room temperature for 2 hr. Eventually, the protein bands were visualized by an enhanced luminol-based chemiluminescent (ECL) reagent. Alliance 4.7 Gel doc (UK) UV Tec software (UK) was used for protein densitometry analysis. β-actin protein was used as the control protein for normalization.

### Statistical analysis

To assess gait scores statistically, a non-parametric Kruskal-Wallis test was employed followed by Dunn’s multiple comparisons, and the data are presented as median with interquartile range. The results of MDA and GSH levels, as well as western blotting, are expressed as mean±standard deviation (SD). Statistical analysis was performed using one-way analysis of variance (ANOVA) followed by the Tukey-Kramer test for multiple comparisons. P-values less than 0.05 were considered statistically significant.

## Results

### Effects of ZEA on the gait abnormalities induced by ACR in Wistar rats

**Figure 1:**
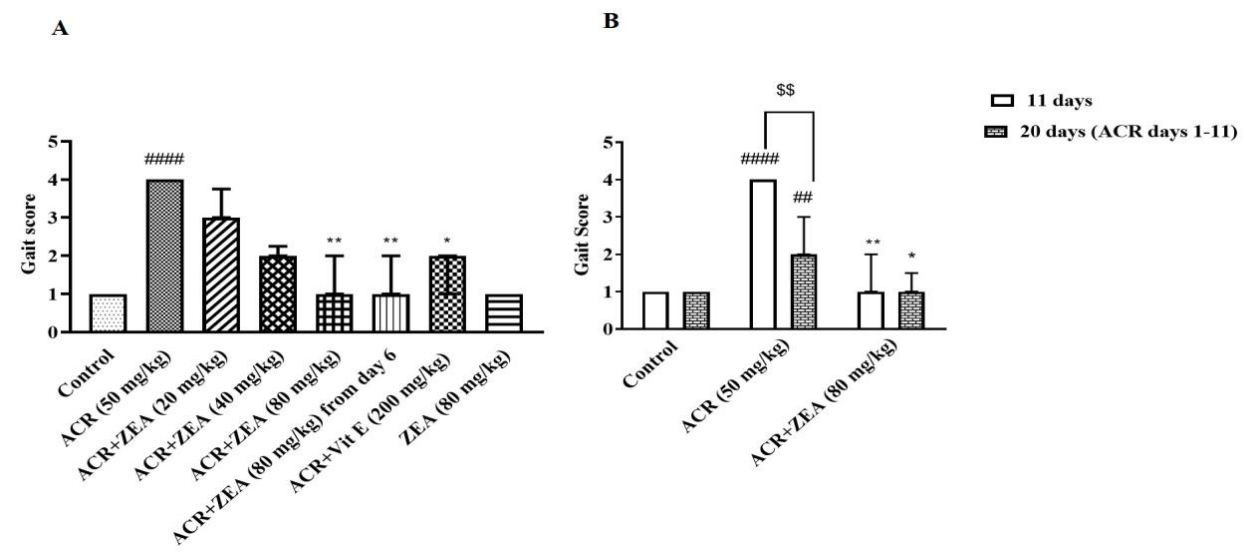
Effects of ZEA on the gait abnormalities induced by ACR in male Wistar rats. A: ACR (50 mg/kg) and vitamin E (200 mg/kg) were administered intraperitoneally and ZEA (20, 40, and 80 mg/kg) by gavage. The behavioral index was assessed after 11 days of administration. B: ACR (50 mg/kg) was administered intraperitoneally and ZEA (80 mg/kg) was administered by gavage to animals. The behavioral index was measured after 11 and 20 days. In both graphs A and B, the data are presented as median with interquartile range (n=6). Kruskal-Wallis non-parametric tests, Dunn’s multiple comparison test, and Mann-Whitney test were used to examine the statistical difference. ###p<0.001 and ##p<0.01 in comparison with the control group; **p<0.01 and *p<0.05 in comparison with the ACR group; $$p<0.01 comparing the groups on days 11 and 20. Acrylamide (ACR), Vitamin E (Vit E), and Zeaxanthin (ZEA).

In Figure 1, it is evident that the administration of ACR (50 mg/kg, i.p.) over 11 days led to the gradual onset of gait abnormalities, as indicated by typical signs of ACR-induced behavioral neurotoxicity (p<0.001 vs control group). Animals that received both ZEA (80 mg/kg, i.g.) and ACR showed a notable enhancement in gait scores in comparison to the group that only received ACR (p<0.01) (Figure 1A). Treating rats with ZEA (80 mg/kg, i.g.) alone did not change the gait score in rats compared to the control group. Administration of vitamin E (200 mg/kg, i.p.) plus ACR alleviated gait abnormalities compared to the ACR group (p<0.05). Rats that received ACR from day 1 to 11 concomitantly with ZEA (80 mg/kg, i.g.) on the last 6 days, had significantly improved gait scores in comparison with the ACR-treated animals (p<0.01).

The group that underwent an 11-day treatment of ACR and was subsequently evaluated on day 20 showed improved gait abnormalities compared to the animals that received ACR for 11 days and were assessed on day 11 (p<0.01) (Figure 1B). Furthermore, to assess the ZEA effect in the longer run, ZEA (80 mg/kg, i.g.) was administered to rats in another group from day 6 to 20 that received ACR for 11 days. The locomotion index of these animals was significantly better compared to others that received ACR for the initial 11 days and then received vehicles from days 6 to 20 (p<0.05). 

### Effects of ZEA on lipid peroxidation induced by ACR

**Figure 2:**
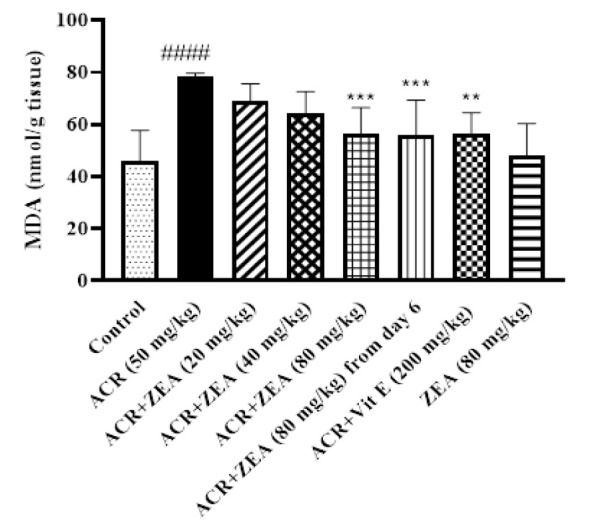
Effects of ZEA and ACR on the MDA level of brain cortical tissue. ACR (50 mg/kg) and vitamin E (200 mg/kg) were administered intraperitoneally and ZEA (20, 40, and 80 mg/kg) by gavage. The data is reported as Mean±SD (n=6). ANOVA test and Tukey-Kramer post-test were used to investigate the statistical difference. ####p<0.0001 in comparison with the control group; and ***p<0.001, and **p<0.01 in comparison with the ACR group. Acrylamide (ACR), Malondialdehyde (MDA), Vitamin E (Vit E), and Zeaxanthin (ZEA)

The extent of lipid peroxidation induced by ACR was assessed by measuring the MDA level in the cerebral cortex of animals. Exposure of rats to ACR caused a significant increase in the MDA levels compared to the control group (p<0.0001). Administration of ZEA (80 mg/kg) decreased MDA levels in the cerebral cortex in comparison to the ACR-treated animals (p<0.001) (Figure 2). Furthermore, administration of 80 mg/kg of ZEA starting on day 6 resulted in decreased MDA levels in rats that were exposed to ACR (p<0.001). Considerable outcomes were noted when rats were simultaneously administered with ACR and vitamin E. (p<0.01 vs the ACR group). Administering ZEA alone did not significantly change the MDA levels compared to the control group. 

### Effect of ZEA on GSH depletion induced by ACR

**Figure 3:**
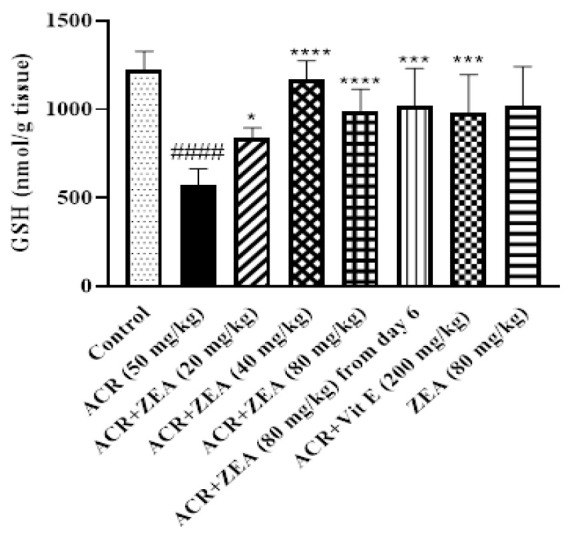
Effects of ZEA and ACR on the GSH level of brain cortical tissue. ACR (50 mg/kg) and vitamin E (200 mg/kg) were administered intraperitoneally and ZEA (20, 40, and 80 mg/kg) by gavage. The data is reported as Mean±SD (n=6). ANOVA test and Tukey-Kramer post-test were used to investigate the statistical difference. ####p<0.0001 in comparison with the control group; ****p<0.0001, ***p<0.001, and *p<0.05 in comparison with the ACR group. Acrylamide (ACR), glutathione (GSH), Vitamin E (Vit E), and Zeaxanthin (ZEA).

Administration of ACR resulted in a significant decline in GSH content in the cerebral cortex of rats (p<0.0001 vs control group) (Figure 3). Treatment with ZEA (20-80 mg/kg) significantly increased GSH levels in comparison to the ACR group (p<0.05 for ZEA 20 mg/kg and p<0.0001 for ZEA 40 and 80 mg/kg). Supplementing with ZEA (80 mg/kg, starting from day 6) in rats exposed to ACR demonstrated a comparable enhancement in GSH content (p<0.001 vs ACR group). Vitamin E (200 mg/kg) manifested its antioxidant properties against ACR toxicity via augmenting GSH (p<0.001) compared to the ACR group. The administration of ZEA 80 mg/kg alone did not induce any changes in the levels of GSH when compared to the control animals.

### Effect of ZEA on neuroinflammation induced by ACR

**Figure 4:**
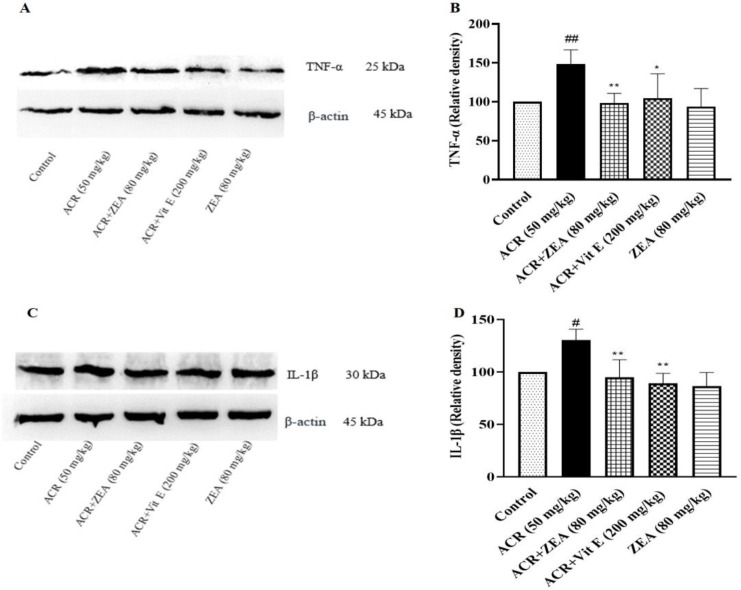
Effect of ZEA and ACR on the TNF-α and IL-1β levels of brain cortical tissue. A, C: Specific bands related to the level of TNF-α and IL-1β, which were examined by Western blotting. B: TNF-α and IL-1β protein levels data by densitometric analysis. The data are expressed as mean±SD (n=4). ANOVA test and Tukey-Kramer post-test were used to investigate the statistical difference. ##p<0.01 and #p<0.05 in comparison with the control group; and **p<0.01 and *p<0.05 in comparison with the ACR group. Acrylamide (ACR), Interleukin-1β (IL-1β), Tumor necrosis factor- α (TNF-α), Vitamin E (Vit E), and Zeaxanthin (ZEA).

In the current study, levels of TNF-α and IL-1β proteins as pro-inflammatory markers were evaluated. In groups treated with ACR, there was a significant increase in the levels of TNF-α and IL-1β when compared to the control group (p<0.01 and p<0.05, respectively). Administration of ZEA 80 mg/kg resulted in a considerable decline in TNF-α and IL-1β levels in comparison with the ACR group, both with a p-value <0.01. In the vitamin E group, reduced levels of TNF-α and IL-1β were observed in comparison to the ACR group (p<0.05 and p<0.01 respectively). On the other hand, ZEA alone did not significantly affect TNF-α and IL-1β expression compared to the control animals (Figure 4). 

### Effect of ZEA on apoptosis induced by ACR

**Figure 5:**
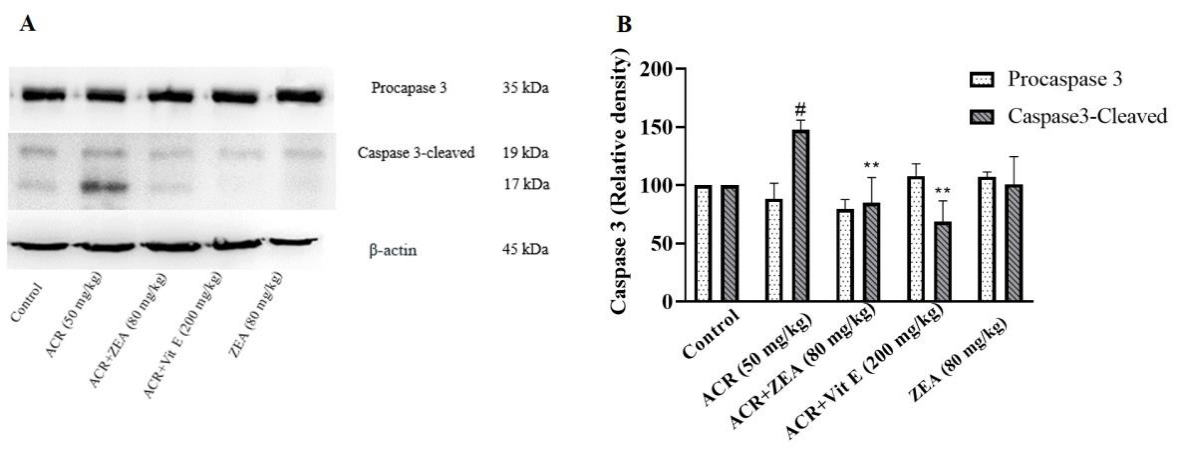
Effect of ZEA and ACR on the caspase-3 (pro and cleaved) levels of brain cortical tissue. A: Specific bands related to the level of caspase-3 (pro and cleaved), which were examined by Western blotting. B: caspase-3 (pro and cleaved) protein level data by densitometric analysis. The data are expressed as mean±SD (n=4). ANOVA test and Tukey-Kramer post-test were used to investigate the statistical difference. #p<0.05 in comparison with the control group; and **p<0.01 in comparison with the ACR group. Acrylamide (ACR), Vitamin E (Vit E), and (ZEA) Zeaxanthin

No significant change in procaspase-3 protein expression was observed in animals that received ACR in comparison to the control group, as indicated by densitometric analysis. However, an increase in caspase- 3-cleaved protein levels in the ACR group was detected (p<0.05 vs the control). Administration of ZEA (80 mg/kg) along with ACR lowered the levels of caspase-3-cleaved compared to the ACR group (p<0.01). The reduction in the quantity of this protein was also apparent following administration of vitamin E (p<0.01 vs the ACR group) (Figure 5). 

## Discussion

This study investigated the protective effects of ZEA against ACR-induced neurotoxicity in rats. Results showed that ACR administration (50 mg/kg, intraperitoneal injection, for 11 days) led to significant motor dysfunction and induced oxidative stress and inflammation in the cortical tissue of the brain. 

However, when ZEA was administered at doses of 20, 40, and 80 mg/kg via oral gavage, starting either from day 1 or day 6 of ACR injection, the most effective dose was found to be 80 mg/kg. This dose, regardless of the timing of administration, effectively reversed the changes induced by ACR, suggesting its potential to mitigate ACR-induced neurotoxicity. It was observed that ACR is widely produced in deep-fried and cooked foodstuffs (Erkekoglu and Baydar, 2014). Previous studies have shown that ACR induces neurotoxicity in mammals’ nervous systems (central and peripheral) and results in weight loss, skeletal muscle weakness, and ataxia (Krishnan and Kang, 2019). Some investigations suggested that neuron degeneration and cellular macromolecule damage might be the underlying mechanisms of motor impairment following ACR neurotoxicity (Kumar et al., 2018; LoPachin et al., 2012).

In line with previous studies, our team observed that administration of ACR (50 mg/kg, i.p., for 11 days) led to weight loss (data not shown), gait abnormality, and dragging hind limbs during walking in rats. Another document has also reported that exposing rats to ACR for 10 days caused paralysis in the majority of the rats (Shukla et al., 2002). Conversely, administering ZEA at a dosage of 80 mg/kg in various protocols (initiated from either day 1 or day 6 of ACR injection) resulted in reduced limb paralysis in rats. To assess the therapeutic potential of ZEA, animals were given this compound starting 6 days after the initiation of ACR administration, with exposure continuing until either day 11 or day 20. Intriguingly, the results demonstrated a significant reduction in motor disorders in both protocols, thus confirming the therapeutic efficacy of ZEA.

The research projects demonstrated that intraperitoneal injection of ACR (50 mg/kg) for 11 days to rats damaged brain cortical tissue, elevated MDA amount, and attenuated GSH level (Ghasemzadeh Rahbardar et al., 2021). 

In the current study, ZEA administered at doses ranging from 20 to 80 mg/kg demonstrated a dose-dependent increase in glutathione (GSH) content, while the highest dose of ZEA (80 mg/kg) significantly reduced MDA levels in the cerebral cortex compared to the groups treated with ACR. Notably, the decrease in MDA levels following administration of ZEA at doses of 20 and 40 mg/kg was not statistically significant, despite significant increases in GSH levels at the same doses. This discrepancy suggests that the observed increase in GSH may not have been sufficient to adequately suppress lipid peroxidation. It also implies that enhancing the activity of other components of the cellular antioxidant machinery, such as antioxidant enzymes, may be necessary to achieve a more substantial reduction in lipid peroxidation. However, further experimentation is warranted to confirm this hypothesis. The protective effects of ZEA through its antioxidant activity, have been highlighted in previous studies. For instance, administration of ZEA at a dose of 80 mg/kg for 6 weeks led to reduced MDA levels and increased antioxidant enzyme activity, such as superoxide dismutase (SOD), in rat models of fatty liver and liver fibrosis diseases (Chamberlain et al., 2009; Xiao et al., 2014). 

Another study demonstrated that adding ZEA to human mesenchymal stem cells exposed to oxidative stress was effective in reducing ROS activity and increasing GSH content (Liu et al., 2017). 

Furthermore, the inflammatory pathways have a chief role in the response to ACR-induced microglial neuroinflammation (Zong et al., 2019). The obtained data of the current work exhibited that receiving ACR (50 mg/kg, 11 days, i.p.) significantly increased the amount of inflammatory factors (TNF-α and IL-1β) in brain cortical tissue in comparison with the control samples. In another study, administration of ACR (20 mg/kg, 30 days, p.o.) to rats elevated the levels of TNF-α and IL-1β in the cerebral cortex (Goudarzi et al., 2019). It was also reported that ACR (20 mg/kg, 4 weeks) exposure increased the expression of TNF-α and IL-1β in the brain of Swiss albino mice (Santhanasabapathy et al., 2015). ZEA 80 mg/kg in the current study significantly reduced TNF-α and IL-1β levels in brain tissue in comparison to the ACR group. Anti-inflammatory properties of ZEA have been illustrated previously in different studies and tissues. Oral administration of 5-250 mg/kg meso-ZEA for 5 days, reduced TNF-α and IL-1β levels, resulting in less pain and inflammation in mice paws (Firdous et al., 2015). Other researchers reported that doses of ZEA alone or alongside lutein were able to suppress TNF-α and IL-1β levels in different tissues (El-Akabawy and El-Sherif, 2019; Ramkumar et al., 2013). When ZEA 50 mg/kg was administered for 14 days, the levels of TNF-α and IL-1β in the hippocampus and subsided depression and anxiety symptoms were lowered (Zhou et al., 2017).

Apoptosis has been identified as a key factor in the development of a variety of brain disorders. The activation of caspases is a powerful predictor of apoptosis (Kekre et al., 2005). Our findings revealed a significant increase in the levels of cleaved caspase-3 protein following ACR administration. In several previous studies, ACR has been shown to induce apoptosis by affecting various caspase proteins, including caspase-3, caspase-8, and caspase-9, as well as other proteins involved in the apoptosis pathway. For instance, in one study, rats orally administered with ACR at a dosage of 50 mg/kg for 11 days, exhibited significantly elevated levels of caspase-3 and caspase-9 in brain tissue compared to the control group (Tabeshpour et al., 2020). In another study, it was discovered that ACR (20 and 40 mg/kg, i.p.) induced apoptosis in the rat spinal cord by increasing the levels of Bax and procaspase-3 apoptotic proteins while decreasing the anti-apoptotic protein Bcl-2 (Li et al., 2006). According to the findings of the present study, administration of ZEA (80 mg/kg, 11 days, i.p.) with ACR significantly reduced the level of caspase-3 protein in cortical tissue compared to the ACR group. Previously, anti-apoptotic properties of ZEA have been reported. ZEA markedly lowered the level of caspase-3-cleaved protein in the hippocampus and recovered cognitive deficit in diabetic rats (Zhou et al., 2017). Also, exposure to ZEA reduced the levels of p-GSK-3β, Bax and caspase-3-cleaved proteins in mesangial cells in a high-sugar environment and showed renoprotective effects (Ying et al., 2017).

Vitamin E exhibited neuroprotective properties in different reports (da Cunha Germano et al., 2023; La Torre et al., 2012). In our study, vitamin E served as a positive control. Compared to the ACR-treated rats, administration of vitamin E reduced movement disorders, decreased MDA levels, increased GSH content, and lowered levels of TNF-α, IL-1β, and cleaved caspase-3 in rat cortical tissue. Vitamin E has consistently demonstrated protective effects against ACR-induced toxicity in various studies (Ghasemzadeh Rahbardar et al., 2021; Tabeshpour et al., 2020). For example, vitamin E (200 mg/kg, i.p.) administration reduced the neurotoxicity induced by 50 mg/kg ACR through antioxidant and anti-apoptosis properties (Ghasemzadeh Rahbardar et al., 2021). 

Interestingly, our research revealed that ZEA at a dose of 80 mg/kg was equally effective as vitamin E in improving gait score, inhibiting oxidative stress and inflammation, and reducing apoptosis in ACR-induced neurotoxicity.

However, our study has several limitations. Incorporating histopathological data could provide further insight into the potential ameliorating effects of ZEA on ACR neurotoxicity. Additionally, we suggest exploring the potential impact of ZEA on other signaling pathways, such as the JAK/STAT pathway and extrinsic or intrinsic apoptosis pathways, to gain a more comprehensive understanding of its mechanisms of action.

In conclusion, ZEA demonstrates both preventive and therapeutic properties in combating ACR-induced neurotoxicity. ZEA effectively mitigates ACR-induced neurotoxic effects and reduces locomotor disorders primarily through its antioxidant, anti-inflammatory, and anti-apoptotic effects in the cerebral cortex of animals.

## Acknowledgment

 The authors are grateful to the Vice Chancellor of Research at Mashhad University of Medical Sciences in Mashhad, Iran for financial help. This article contains information from a Pharm D Thesis.

## Conflicts of interest

The authors have declared that there is no conflict of interest.

## Funding

The authors are grateful to the Vice-Chancellor of Research, Mashhad University of Medical Sciences for financial support (grant number: 971986).

## Ethical Considerations

All animal experiments were carried out according to the rules of Mashhad University of Medical Sciences, Ethical Committee.

## Code of Ethics

 The ethical code for this study is ID REC.PHARMACY.MUMS.IR.1398. 026

## Authors' Contributions

H. H. Conceptualized the study. Z. M. executed the animal experiments, collected and analyzed the data and initially drafted the manuscript. M. G. R. and S. M. supervised the study. Z. M. and M. G. R. and S. M. and H. H. contributed to the revision of the manuscript. All authors have read and approved the final submitted manuscript.
